# The Chronic Indigence of Our Hospitals, with Some Suggestions for Its Remedy

**Published:** 1892-04-02

**Authors:** B. Burford Rawlings


### The Institutional Workshop.

### THE CHRONIC INDIGENCE OF OUR HOSPITALS, WITH SOME SUGGESTIONS FOR ITS REMEDY.

By B. Burford Rawlings.

### I.—A BIRD'S-EYE VIEW.

For many a day the impoverished condition of our hospitals has been a subject of concern to those whom necessity or inclination has made familiar with the somewhat painful details of hospital finance. A statement has been authoritatively published that the deficit of 1890 has been exceeded in 1891. In other words, whatever may be affirmed of certain favoured hospitals, the income of the whole number is a diminishing quantity.

That of itself is a serious matter, but it is only a part of the case, because this statement of a deficit means nothing more than a failure to cover the aggregate cost of carrying on certain work, portions of which a number of independent organisations have set themselves to do, each undertaking just so much as its managers think well, or as the income they are enabled to collect will pay for.

It is manifest that the figures of a deficit thus ascertained, however truly they represent the mere difference between the receipts and the expenditure of a given period, are altogether illusory when we look to them to indicate what relation the income supplied to the hospitals bears to the cost o an ample, but not excessive provision for the legitimate wants of the sick poor, and an enlightened and comprehensive system of medical education. Apparently, nobody is in a position to state positively what the extent of work undertaken by our voluntary hospitals ought to be. Perhaps the suggestion likely to arouse the least opposition is the somewhat crude one that the provision for serious cases, represented, roughly by in-patients, is deficient, while for more trivial cases represented, roughly again, by out-patients, it is excessive.

However this may be, we all know that in reckoning the cost of hospital work no heed is taken of the fact that some important workers, including the most essential of all, the medical and surgical staff, receive no direct remuneration, while others receive only partial and insufficient remuneration. In this dependence upon gratuitous and honorary service, we perceive one indication of hospital indigence.

We need not discuss whether the medical officers are actuated by motives of pure disinterestedness in assuming their laborious functions. We know that very onerous duties are demanded of them, and that these are performed, so far as the patient is concerned, with a thoroughness which is universally acknowledged. Obviously, philanthropy alone could make no real use of our hospitals, and if they were not the indigent institutions they are, we may be sure that for more reasons than one appointments would cease to be honorary in any sense. To remunerate the staff adequately would involve an additional annual expenditure of say £130,000,* and that enormous sum, minus if you please the value of the advantages obtained, represents the contribution of the profession to the cause of hospital relief.

It cannot be doubted that if poverty were not of the essence of our hospital system, we should cease to remunerate the nurses upon a scale scarcely raised above the level of a housemaid's wages. An act of obvious justice is not uncommonly denied an official for no better reason than that it is charitable money which is being disbursed, and if the work and responsibilities of some of the paid officials were adequately appraised, it might be found that not a few are among the largest contributors to the institutions they serve.

*The method of arriving at these figures will be dealt with later.

If, besides this deficit in respect of the actual cost of the work carried on, there is also an insufficiency and an unsuitability of accommodation, then the case is so much the stronger; but were there no money deficit, nor material insufficiency, nor ill-paid and honorary services, the hospitals would still be indigent institutions, working under all the disadvantages indigence entails, fighting a never-ending battle for life, and with the difficulties of an administration charged with complex and delicate details enormously increased in consequence.

The case for the hospitals may be put thus. A person of the poorer classes falls ill. His circumstances may be somewhat better than those of the 200,000 families who, we are told, subsist in London upon incomes of less than £1 per week a-piece, or he may be of that number. In the first case, his means enable him to pay for medical attendance—of a kind. No one could suppose it of the best, or if it were that it comprises all a sick man needs for recovery. The position is not altered should he have been virtuously provident, because we are considering not the victims of ailments easily treated at home or at the provident dispensary, but those graver maladies which incapacitate for a longer or shorter period, and call not only for continuous and skilful treatment, but for good nursing and expensive dietary. Both classes are in precisely similar positions in this respect; they are unable to obtain what they want anywhere but at the voluntary hospitals. Again, a young man wishes to adopt the profession of medicine, and he finds there is no provision for his education except at the voluntary hospitals. That is to say, the whole debt and duty we owe to our sick poor and to medical science is left to be discharged by these institutions, condemned to beg their daily bread and recognised by the State, only in order that they may be rated to the parish.

The assessment varies with the locality. In some parishes the authorities perceive apparently that to levy poor rates upon institutions whose work is designed in the interests of the poor is not altogether a luminous proceeding, and a merely nominal assessment is accordingly made, an agreeable arrangement which the County Council has been lately doing its best to destroy. In others, there is no surrender to sentiment or humanity. The utmost fraction is demanded and the hospitals must obtain many hundreds of pounds every year for the rates' collector before they can turn their attention to providing for the needs of the sick ones lying in their care. As much as £3 or £4 per bed is contributed in some instances, and yet, as if to add insult to injury, the institutions which contribute these enormous sums are disfranchised and unrepresented. Neither at local, County Council, nor Parliamentary elections are their authorities permitted to vote. Could the force of unreason and injustice much farther go?

The annual expenditure of the Metropolitan voluntary hospitals is put in the last Sunday Fund number of the *Lancet* at £606,000, of which we may estimate that two-fifths are supplied by endowments unequally distributed, leaving the remainder to be raised in benefactions composed of subscriptions, donations, and legacies. One institution only, St. Bartholomew's, is fully protected against the anxieties of an impecunious state, though even this splendid hospital, in view of increasing work, is understood not to be averse from receiving additional donations, if only of sufficiently respectable proportions. St. Thomas's has wards closed for want of means to maintain them. Guy's is in like case, and actively appealing for help; the London, that stronghold of leviathan philanthropy, is held by a race of giants who, alas! may yet prove mortal; St. George's in a characteristically quiet way is assimilating the product of a few years of plenty; Middlesex is already selling out some part of the stock a lucky windfall recently enabled it to buy; King's is ever appealing to law to come to the help of medicine; St. Mary's announces forcibly that aid was never more urgently wanted; Westminster tells of a deficiency in receipts, as compared with expenditure, of twenty-five per cent; while University College and Charing Cross vie with undeviating regularity in the presentment of a desperate case. If we turn from the general hospitals to the larger special institutions, we find less debt perhaps, and fewer beds out of use, but a more manifest inadequacy of provision for the classes of sufferers dealt with, which is not remedied, but on the contrary, is aggravated by the active competition of a number of small organisations, some of them of doubtful origin and questionable utility.

It is a principle apparently considered fundamental to English benevolent institutions that they should be "supported by voluntary contributions," and there is an indisposition, almost unreasonable in its disregard of certain obvious facts, to do anything which should tend to render this voluntary assistance less casual and uncertain. The large majority of people do not appear to approach this question seriously. They assume, apparently, that a state of indigence is beneficial, that it induces circumspection, and they are at no trouble to enquire at what wasteful cost, or at what sacrifice of efficiency the tradition is maintained. It would surprise them to be told that the never-ceasing need of money, and the competition with other institutions entail a process in which the difficulties in the way of good management are greatly increased, while a substantial part of the help rendered and not a little of the spirit of philanthropy are allowed to escape.

If we were preparing to write upon the Voluntary System, the phrase oftenest in use, like the author who composed a celebrated chapter on snakes, we should immediately suffer from a dearth of material. There is no system. Our voluntary hospitals are all of independent origin; with scarcely an exception they have been born of the desire of philanthropic individuals, some of whom have made provision for their offspring, while others have been content with the pleasures of parentage, and have left their children a charge upon the good offices of other people. But whether endowed, partly endowed, or altogether impecunious, the institutions are alike sovereigns of themselves, jealous of their individual liberties, proud of their traditions, and possessed of an esprit, natural and altogether admirable.

Of the work performed by each of the several recognised institutions, it would be difficult to speak too highly. Although the report of the Lords' Select Committee is not yet issued, the Chairman and other members have not left us in doubt of their opinions. There are no more perfect hospitals anywhere, is the verdict. But the very highest praise which can be offered is inadequate and unsympathetic if it does not take into serious account the difficulty of doing good work under the disadvantages and disabilities of chronic indigence. If it were a question of considering how each institution does what it has undertaken, we might well be satisfied. It is when we come to consider the work of medical relief as a whole that our doubts grow to certainties, and side by side with a grateful appreciation of all that is being accomplished, a feeling strengthens that the task is too vast, its requirements too complicated, and the interest and issues involved too tremendous, to be undertaken piecemeal.

Consider for a moment what it means that the work of a great public department, for we are contemplating nothing less, should be assumed by a body of independent and voluntary agencies left to obtain the major part of their incomes by begging, and begging in competition, without the power to demand of anybody a single penny, liable to have their supplies curtailed or stopped if they fail to satisfy requirements, however unreasonable, and at the mercy of any ill-natured or ignorant criticism that may be offered. The unreasonableness of many complaints, and which sometimes find their way into print, is incredible. It is well illustrated by a remonstrance recently addressed to a hospital in regard to an out-patient who had attended alone and subsequently fallen ill in the street. The hospital ought, it was said, to see its unattended patients safely home. Sometimes the criticism is nothing more than the expression of a personal desire to punish the institution or some disappointment magnified to a grievance. But if there be shortcomings—and who will deny them?—is that an adequate reason for taking away income, and how is efficiency to be promoted by such punishment? You might as reasonably take away a mechanic's tools and refuse him food in order to improve his workmanship. To state the case thus reduces it to the absurdity it is. Hospital managers will readily admit that in holding the door against the wolf which is always threatening, they must sometimes lose sight of that they would like to keep in view. They often have reason to regret the preoccupation a state of penury involves, and no one could rightly object to the controlling influence of capable opinion. The Government itself is the subject of criticism, enlightened or the reverse, but how, it may be pertinently asked, would government be possible, if everybody who was dissatisfied with some detail of administration were at liberty to refuse payment of taxes?

Consider, too, in this connection that the greater number of benevolent people are largely influenced by sentiment. Compassion is more powerful than a sense of duty. This is proved by the fact that as individuals they give far more than duty, however exacting, could demand. It is strictly consistent with their instincts of benevolence that their gifts should be freely made in relief of personal suffering rather than to promote the general operations of medicine, however beneficent, and the desire for a purely philanthropic action induces them to rebel against the suggestion that an economical aspect attaches to hospital work. To plead for aid upon the ground that the hospital is useful to the community has been shown to be at least ineffective. So far from helping a hospital financially, a too luminous scientific reputation militates against it. The religion of philanthropy is absolute enough to resent the presence of any alien creed, and the searchings of science after more light come to be regarded as somewhat unwarrantable intrusions into regions sacred to suffering and darkness. By all means let the hospitals remain voluntary institutions dependent upon voluntary support, but let the voluntaryism be of a righteous and reasonable kind. Let it not be merely licence to do or leave undone at will. We all claim to be the free inhabitants of a free country, but that does not mean that we have no duties or that we live without laws. The hospitals, and, in a far greater degree, the non-contributing public, may with advantage apply this reasoning to themselves.

